# A New Disc Mandrel

**Published:** 1892-02

**Authors:** 


### A New Disc Mandrel.

One of the most complete disc carriers that we have seen has just been devised by Dr. E. C. Moore, of Detroit, Mich. It consists of a screw about one-fourth of an inch in length, with a grooved head upon one end; a screw collar is made that is tapped to run upon the screw, this collar having a flange the same size as the head of the screw. For mounting the disc of paper, or any other material, the screw is passed through it, the collar is put upon the screw and drawn up tightly upon the disc; the other part of the appliance consists of a screw socket shaft that runs into the engine, and the disc, as described, is simply screwed into this socket shaft, when it is ready for use. One or two socket shafts is enough for any number of the mounted discs, as they are put on or taken off in a moment's time by a turn of the screw either backward or forward, as the case may be.

This is one of the cheapest and most efficient appliances for this purpose we have seen. This appliance will very soon be obtainable through the dental depots.

